# Sustainable valorization of grape pomace peels using NADES: a focus on selective recovery of anthocyanins and flavonols and bioactivity

**DOI:** 10.1007/s00216-025-06164-9

**Published:** 2025-11-01

**Authors:** Andrea Palos-Hernández, Miguel del Nogal Sánchez, M. Yolanda Gutiérrez Fernández, José Luis Pérez-Iglesias, Celestino Santos-Buelga, Ana M. González-Paramás

**Affiliations:** 1 Grupo de Investigación en Polifenoles (GIP-USAL), Facultad de Farmacia, Universidad de Salamanca, Campus Miguel de Unamuno S/N, 37007 Salamanca, Spain; 2 Departamento de Química Analítica, Nutrición y Bromatología, Universidad de Salamanca, Salamanca, Spain; 3 Departamento de Informática y Automática. Escuela Politécnica Superior de Zamora, Universidad de Salamanca, Zamora, Spain

**Keywords:** Natural deep eutectic solvents, Response surface methodology, Anthocyanins, Flavonols, Grape pomace

## Abstract

This study investigates novel approaches for the green extraction of polyphenols from grape pomace. Sixteen natural deep eutectic solvents (NADES) systems, based on either choline chloride or sucrose as hydrogen bond acceptors (HBAs) and one of tartaric, malic, lactic, oxalic, or citric acids, or 1,2-propylene glycol as hydrogen bond donors (HBDs), were evaluated. Response surface methodology (RSM) was applied to optimize the extraction parameters, namely water content and extraction time, for maximizing total anthocyanins and total flavonols content using different combinations of choline chloride and lactic acid. Optimal conditions were found to be not more than 25% (*v/v*) of water and an extraction ratio of 30 mL/g dw. Overall assessment of the results pointed at the system ChCl:lactic acid, 1:2 containing 25% water (CL25) as a satisfactory compromise alternative for both anthocyanin and flavonol extractions. Nevertheless, marked differences were found in the affinity of different NADES towards distinct phenolic compounds, which could be exploited for the rational selection of the most suitable solvent depending on the phenolic profile of the matrix or when searching for particular target compounds. In addition, the extracts obtained with distinct NADES showed notable antioxidant activity and, unlike conventional methanolic extracts, also exhibited antimicrobial effects attributable to the extraction solvent itself, highlighting their potential for food-related applications.

**Graphical Abstract:**

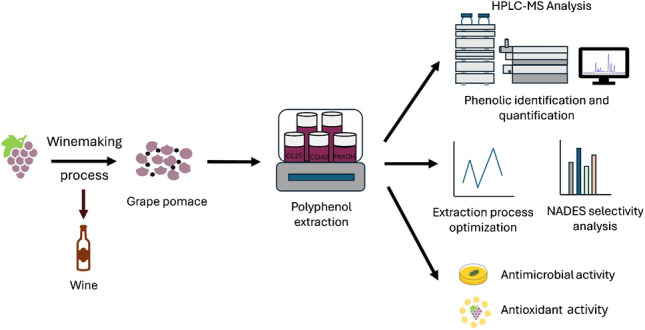

## Introduction

Grapes (*Vitis *spp.) are succulent berries cultivated worldwide on approximately 7.4 million hectares, primarily for the winemaking industry (≈ 90%) [1–3]. This sector generates substantial waste, notably grape pomace, a biodegradable solid by-product whose yield, typically 20–30% of the original grape mass, varies with vinification method and cultivar [4]. This large amount of waste has a huge environmental impact alongside the economic cost of not treating or reusing it. Among the potential strategies, the valorization of these by-products through the extraction of bioactive compounds has received considerable attention.

Within the various families of bioactives, phenolic compounds have particular interest due to their recognised bioactive properties, including antibacterial, anti-inflammatory, antioxidant, and anti-tumour effects [5]. During the winemaking process, only 30–40% of the phenolic compounds are extracted into wine [6]. As a result, grape pomace remains a rich source of phenolics, whose composition is strongly influenced by climatic conditions, grape variety, and the specific winemaking process used [7]. The phenolic compounds present in grape pomace can be classified into three main groups: phenolic acids (benzoic and hydroxycinnamic acids), flavonoids (catechins, flavonols, and anthocyanins), and tannins. Among the anthocyanins, the 3-glycosides, 3-acetylglycosides, and 3-*p*-coumaroylglycosides of malvidin, peonidin, delphinidin, petunidin, and cyanidin are the compounds most found in red grapes [8]. In the case of flavonols, quercetin and kaempferol derivatives are the predominant constituents [9].

Grape pomace biomass has been widely studied as a source for the extraction of phenolic compounds, using organic solvents such as methanol, ethanol, or acetone, both directly or in combination with novel techniques. These include microwave-assisted extraction [10], solid–liquid extraction [11], high-voltage electrical discharges [12], pressurized-assisted extraction [13], and enzyme-assisted extraction [14]. The use of these organic solvents has been questioned due to their environmental impact and the safety issues associated with such substances, which should be removed before the extracts can be used for industrial applications. As a result, there is an increasing interest in developing greener, cleaner, safer, and easier-to-implement extraction techniques that enable the sustainable valorization of agro-industrial waste [15].

Natural deep eutectic solvents (NADES) have emerged as a promising green alternative for the extraction of phenolic compounds. These solvents typically consist of hydrogen-bond acceptors (HBA), such as non-toxic quaternary ammonium salts (e.g. choline chloride), and naturally derived uncharged hydrogen-bond donors (HBDs), including amines, sugars, alcohols, and carboxylic acids [16]. These solvents have been applied to phenolic extraction from different plant and food matrices, such as *Catharanthus roseus [17],* blueberry [18], *Platycladi cacumen* [19], olive [20], *Lippia citriodora* [21], *Dracocephalum kotschyi* [22] or mangosteen [23]. Choline chloride (ChCl) is the most commonly used hydrogen bond acceptor, while the hydrogen bond donor varies and plays a critical role in interacting directly with the target compound, significantly affecting extraction efficiency. For anthocyanins, acidic conditions enhance recovery, as the flavylium ion is stabilized at low pH, improving both extraction and stability. Consequently, NADES containing organic acids such as citric and malic acids—due to their high polarity and acidity—usually yield higher anthocyanin recoveries [24–26]. For flavonols, alcohol-based HBDs have been proposed. However, flavonoids themselves can function as HBDs, leading to competitive interactions with chloride anions. Therefore, the design of an effective alcohol-based HBD for flavonol extraction requires adequate spacing between HBD groups and minimal branching. Several authors have reported good results using lactic acid [19, 21, 27, 28], while others, such as Cui et al. [29] and Cvjetko et al. [30], obtained satisfactory results using oxalic acid and 1,4-butanediol as HBD.

Only a limited number of studies have explored green extraction methods using NADES for the recovery of phenolic compounds from grape pomace. Among these, several have reported improved anthocyanin extraction yields using choline chloride-based NADES in combination with malic acid [24], oxalic acid [31], maltitol [32], fructose [32], or citric acid [25], while betaine-based NADES have been employed by Punzo et al. [33] to recover flavonoids from grape peel obtained from the pomace for potential applications in skin care.

Given the limited research available, this study aims to develop and optimize a greener extraction method for simultaneously recovering anthocyanins and flavonols from grape pomace peels using NADES.

## Materials and methods

### Samples, standards, and reagents

Choline chloride (ChCl ≥ 98.0% purity), citric acid monohydrate (≥ 99.0% purity), DL-tartaric acid (99.0%), DL-lactic acid (90.0%), oxalic acid dihydrate (≥ 99.5% purity), DL-malic acid (≥ 98.0% purity), quercetin dihydrate (97%), and phosphate-buffered saline (PBS) were purchased from Sigma-Aldrich (Madrid, Spain); and 1,2-propilene-glycol (99.0%), sucrose (99%), and hydrochloric acid (37%) were from Panreac (Barcelona, Spain). Malvidin-3-*O*-glucoside was purchased from Extrasynthese (Genay, France), and quercetin dihydrate (97% purity) from Alfa Aesar (MA, USA).

All chemicals for HPLC analyses (methanol, formic acid, trifluoroacetic acid (TFA), and acetonitrile (ACN)) were of analytical grade and purchased from Prolabo VWR (Fontenay-sous-Bois, Francia) and Panreac (Barcelona, Spain). Ultrapure water was obtained from a Milli-Q Gradient water purification system (Millipore, Billerica, MA, USA).

Culture media: tryptic-soy agar (TSA) and Müeller-Hinton broth (MH) were purchased from Scharlau (Sentmenat, Spain).

The Tempranillo grape pomace used in this study was provided by a local winery in Zamora, Spain, in 2020 and kept frozen (–30 °C) until use.

### NADES preparation

NADES used in this study were prepared by the heating and stirring method proposed by Savi et al. [34]. The components were weighed in a capped flask, according to the mole ratio given in Table 1, and mixed with agitation on a heating plate at 50 °C until a homogeneous, transparent liquid was obtained.

**Table 1:** Composition of the prepared natural deep eutectic solvents

Abbreviation	Components		Mole ratio	% Water
	HBA	HBD		
CT40	Choline chloride	Tartaric acid	1:1	40
CT60				60
CP25		1,2-Propylene glycol	1:2	25
CP40				40
CM25		Malic acid	1:1	25
CM40				40
CL25		Lactic acid	1:2	25
CL40				40
CO25		Oxalic acid	1:1	25
CO40				40
CC25		Citric acid	1:1	25
CC40				40
SC25	Sucrose	Citric acid	1:1	25
SC40				40
SL25		Lactic acid	1:4	25
SL40				40

### Phenolic compounds extraction. NADES screening

The grape pomace sample was separated into seeds and skins. Subsequently, the skins were subjected to freeze-drying and grinding in order to obtain a homogeneous powder. The six different choline-based NADES and two sucrose-based NADES described in Table 1 were tested for their effectiveness in extracting total anthocyanins and flavonols from the grape peels obtained from the pomace. It is known that the water content can significantly impact the efficiency of extraction [27]. Therefore, water proportions ranging from 25 to 60% (w/w) were tested. Approximately 2 mg of powder was extracted with 40 mL of each NADES. The mixture was placed in a shaker at 40 °C and 210 rpm for 1 h. The extracts were then centrifuged at 14,400 × *g* for 10 min, and the resulting supernatants were filtered through a 0.45-µm hydrophilic PVDF (polyvinylidene fluoride) membrane. In order to evaluate the performance of the selected NADES, an analysis was carried out by HPLC, for which the extracts were diluted to twice their volume with 0.1% TFA:ACN solution (80:20) for anthocyanin analysis or 0.1% formic acid:ACN (85:15) for other phenolics. The extraction efficiency was then compared with that of a conventional solvent consisting of an acidified aqueous solution of methanol-hydrochloric acid 0.5 N, 95:5 (*v/v*) (MET).

Finally, the nine most effective solvents were selected for a comprehensive analysis that included recovery rates, selectivity, and process optimization. The analysis was carried out in triplicate to ensure the reliability of the results.

### HPLC-DAD-MS analysis

Analysis was performed using a Hewlett-Packard-1200 HPLC-DAD chromatograph (Agilent Technologies, Palo Alto, CA, USA) equipped with a binary pump and a diode array detector (DAD) connected to an HP Chem Station (rev.A.0504) for data processing. The HPLC was connected via the DAD cell output to an API-3200 Qtrap (Applied Biosystems, Darmstadt, Germany) mass spectrometer (MS) equipped with an ESI source and a triple quadrupole-ion trap mass analyser, controlled by the Analyst 5.1 software.

*Anthocyanin* separation was performed on a 5-μm AQUA® C18 150 × 4.6 mm column (Phenomenex®, Torrance, CA) thermostatted at 35 °C. The mobile phase comprised 0.1% TFA in water (A) and HPLC grade ACN (B), establishing the following gradient: isocratic 10% B for 5 min, 10–15% B during 15 min, isocratic 15% B over 5 min, 15–18% B for 5 min, and 18–35% B during 20 min, using a flow rate of 0.5 mL/min. Double online detection was employed in the DAD, setting the preferred wavelength at 520 nm and operating the MS in the positive ion mode. Spectra were recorded between *m/z* 100 and *m/z* 1500. Zero-grade air (40 psi) was used both as a nebulizer gas (40 psi) and as a turbo gas (600 °C) for solvent drying (50 psi). Nitrogen was used as curtain gas (100 psi) and collision gas (high). Both quadrupoles were configured to operate at unit resolution, and the MS detector was programmed to execute two consecutive analyses: a high-sensitivity full scan (Enhanced MS, EMS), followed by an enhanced product ion (EPI) analysis to acquire the fragmentation pattern of the parent ion. The EMS mode parameters were set up as follows: entrance potential (EP) 7.5 V, declustering potential (DP) 41 V, and collision energy (CE) 10 V. The EPI mode parameters are as follows: EP 7.5 V, DP 41 V, EP 7.5 V, and collision energy spread (CES) 0 V.

*Flavonols and phenolic acids* were separated using an Agilent Poroshell 120 EC-C18 column (150 mm × 4.6 mm, 2.7 μm) thermostatted at 35 °C. The mobile phase consisted of 0.1% formic acid in water (A) and HPLC-grade ACN (B). The following elution programme was applied at a flow rate of 0.5 mL/min: isocratic 85% A during 5 min, from 85 to 8% A for 5 min, from 80 to 65% A for 10 min, from 65 to 50% A for 10 min, from 50 to 60% A for 2 min, an isocratic gradient of 60% A for 5 min, followed by a return to the initial condition and equilibration. Double online detection was conducted in the DAD, setting the preferred wavelengths at 330 and 360 nm and operating the MS in the negative ion mode. Spectra were recorded between *m/z* 100 and *m/z* 900. Zero-grade air was used both as a nebulizer gas (40 psi) and as a turbo gas (400 °C) for solvent drying (40 psi). Nitrogen was used as curtain gas (100 psi) and collision gas (medium). Both quadrupoles were configured to operate at unit resolution, and the MS detector was programmed to execute two consecutive analyses: a high-sensitivity full scan (Enhanced MS, EMS) followed by an Enhanced Product Ion (EPI) analysis to acquire the fragmentation pattern of the parent ion. The EMS mode parameters were set up as follows: entrance potential (EP), 6 V; declustering potential (DP), 50 V; and collision energy (CE), 10 V. The EPI mode parameters were: EP, 6 V; DP, 50 V; CE, 10 V; and collision energy spread (CES), 0 V.

The identification of phenolic compounds was performed on the basis of their retention time, UV–vis and mass spectra, as well as a comparison with our data library, available standards and/or literature data. Quantification was carried out from the areas of the chromatographic peaks recorded at 520 and 360 nm (for anthocyanins and flavonols, respectively), using seven-point calibration curves (0–100 mg/L) prepared with commercial standards of malvidin-3-*O*-glucoside (Mv3glc) and quercetin. The results were expressed in milligrams per 100 g of dry weight. Although the chromatograms also revealed the presence of phenolic acids, their identification was limited to a qualitative level, as the present study focused specifically on the optimization of the extraction of anthocyanins and flavonols.

### NADES selectivity

The selectivity of the NADES for the extraction of anthocyanins and flavonols was assessed by calculating the proportion of each main quantified compound relative to the total content of quantified anthocyanins or flavonols, respectively.

$$\mathrm{Selectivity}\;\left(\%\right)=\frac{\mathrm{recovery}\;\mathrm{target}\;\operatorname{compound}}{\mathrm{total}\;\mathrm{anthocyanins}\;\mathrm{or}\;\mathrm{flavonols}\;}\times100$$

For the statistical analysis, GraphPad Prism 10.2.2 software (GraphPad Software, Boston, MA, USA) was used. Selectivity *p*-values were analysed by one-way analysis of variance (ANOVA) with Dunnett’s multiple comparisons test. All experiments were performed in triplicate. *p*-values were considered as follows: 0.12 (ns), 0.033 (*), 0.002 (**), < 0.001 (***).

### Optimization of the extraction methodology by response surface methodology (RSM)

Response surface methodology (RSM) was applied to determine the optimal levels of two factors: water content (%) and the liquid-to-solid ratio (mL/g d.w.) regarding the yields of anthocyanins and flavonols, expressed as a function of the predominant compound for each group (Mv3glc and quercetin, respectively). Experiments were designed according to the “Circumscribed Central Composite” (CCCD) [22], using a 2^2^ factorial (4 experiments) and star (4 experiments) design with four central points. The ranges for the studied factors were selected based on literature data and preliminary results. The experimental sequence was randomized to minimize the influence of unexpected variations due to external factors. Four models were evaluated for data fitting: linear (including only the factors), quadratic 1 (including factors and their squares), quadratic 2 (including factors and their interactions), and quadratic 3 (including the factors, their squares, and interactions). Thus, for example, the equation for model 3 is as follows:

$$Y={\beta }_{0}+{\beta }_{1}{X}_{1}+{\beta }_{2}{X}_{2}+{\beta }_{11}{{X}_{1}}^{2}+{\beta }_{22}{{X}_{2}}^{2}+{\beta }_{12}{X}_{1}{X}_{2}$$

where *Y* is the value of the response variable predicted by the model, *X*_i_ and *X*_j_ are the selected independent factors, *β*_0_ is a constant, *β*_1_ and *β*_2_ the linear coefficients, *β*_11_ and *β*_22_ the quadratic coefficients, and β_12_ is the interaction coefficient between the tested factors. Finally, the relationship between independent variables and responses was examined using an analysis of variance (ANOVA) test with a confidence level of 95%.

The Unscrambler software (v 10.2, CAMO Software Inc., Woodbridge, NJ, USA, 2012) was used for the ANOVA to obtain the polynomial mathematical model, which was established to describe the extraction process parameters for total anthocyanins and flavonols. The determination coefficient (*R*^2^) and the model *p*-value were used to assess the predictive capability of the model.

### Total phenolic content (TPC)

The total phenolic content (TPC) of the NADES extracts was determined by the Folin-Ciocalteu method [35]. Briefly, 10 µL of each extract was mixed with 10 µL of Folin-Ciocalteu reagent, followed by the addition of 200 µL of 7.5% Na_2_CO_3_. The volume was then completed to 500 µL with deionized water. Blanks were prepared in a similar way, substituting the extract with 100 µL of water plus each solvent. After standing in the dark for 1 h, absorbance was measured at 750 nm. Phenolic concentrations were determined from a gallic acid calibration curve and expressed as mg gallic acid equivalents (GAE) per mL of extract. All analyses were performed in triplicate.

### Antioxidant activity

#### Ferric reducing ability power (FRAP)

Ferric reducing antioxidant power was determined as described by Benzie and Strain [36] with slight modifications. The FRAP reagent was composed of a 10 mM TPTZ solution, which was dissolved in 40 mM HCl, 20 mM iron trichloride (FeCl₃·6H₂O), and acetate buffer (300 mM, pH 3.6), at a volume ratio of 1:1:10. For each assay, 250 µL of the reagent was mixed with 10 μL of sample and incubated at 37 °C for 6 min, after which absorbance was measured at 593 nm. In addition, blanks containing FRAP reagent and each NADES solvent were analysed. The difference between the sample and blank absorbances was utilized for calculation. Results were obtained by interpolating the mean absorbance of three replicates on a Trolox calibration curve (30–1000 μM, *R*^2^ = 0.9986). Data were expressed as mM of Trolox equivalents (TE).

#### ABTS radical scavenging assay

The ABTS precursor (2,2′-azinobis(3-ethyl-benzothiazoline-6-sulphonate)) was prepared at a concentration of 7 mM, and 2.45 mM potassium persulphate was added as an oxidizing agent for the generation of the radical. The solution was stored in the dark for 16 h and diluted with a sufficient volume of phosphate-buffered saline (PBS) at pH 7.4 to give an absorption of 0.7 ± 0.02 at 734 nm. Subsequently, 7 μL of each extract was mixed with 200 μL of diluted ABTS solution. After 4 min, the absorbance was measured at 734 nm. Blanks were prepared by mixing ABTS reagent with each NADES solvent; the difference between the sample and blank absorbance values was used for calculation. The mean absorbance from three replicates was converted to millimolar Trolox equivalents (mM TE) using a standard calibration curve of Trolox in water (30–1000 μM; R2 = 0.9974).

### Antimicrobial activity

Two bacterial strains acquired from American Type Culture Collection (ATCC, Manassas, VT, USA) and the Spanish Type Culture Collection (Valencia, Spain) were used in this work: three Gram-positive strains (*Staphylococcus aureus* ATCC 25923, *Bacillus subtilis* CECT 35, and *Listeria innocua* CECT 910) and two Gram-negative strains (*Escherichia coli* ATCC 25922 and *Proteus mirabilis CECT* 170). The selection of bacteria was based on their pathogenicity and the risk they pose to food safety. *E. coli*, *P. mirabilis*, *B. subtilis*, and *S. aureus* were included due to their relevance as human pathogens and their potential transmission through the food chain. *L. innocua* was selected because of its similarity to *L. monocytogenes*, a high-risk foodborne pathogen. It is also widely used as an indicator of the effectiveness of disinfection processes. All bacterial strains were routinely grown on tryptic soy agar (TSA).

Bacterial susceptibility to the extracts was assessed by the broth microdilution method [37], with slight modifications. In brief, bacterial inocula were prepared by suspending bacteria in 0.85% (*w/v*) NaCl and adjusting the turbidity to 0.5 McFarland, corresponding to approximately 5 × 10⁶ CFU/mL. Seven serial dilutions of each extract were prepared, then incubated at 37 °C for 24 h. Afterward, 2 µL of each dilution was placed in a petri dish with Mueller-Hinton medium and incubated for a further 48 h at 37 °C to confirm bacterial growth or inhibition. All assays were performed in triplicate.

Three controls were used alongside the extracts: a sterility control (medium only), a growth control (medium with bacterial suspension), and a negative control (ampicillin with bacterial suspension). Results were expressed as minimum inhibitory concentrations (MICs), defined as the lowest concentrations of an inhibitor that inhibit visible microbial growth after 48 h incubation.

## Results and discussion

### Phenolic composition

**Fig. 1:**
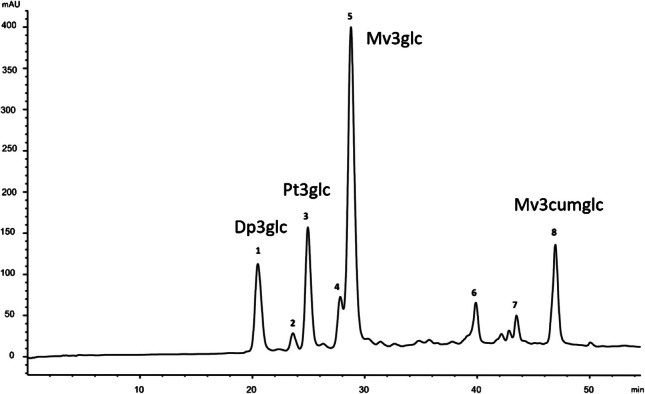
Characteristic HPLC chromatogram of the extracts obtained from the grape peels obtained from the pomace recorded at 520 nm. Peak numbers are identified in Table 2. Abbreviations: Dp3glc, delphinidin 3-O-glucoside; Pt3glc, petunidin 3-O-glucoside; Mv3glc, malvidin 3-O-glucoside; Mv3cumglc, malvidin 3-O- coumaroylglucoside

**Fig. 2:**
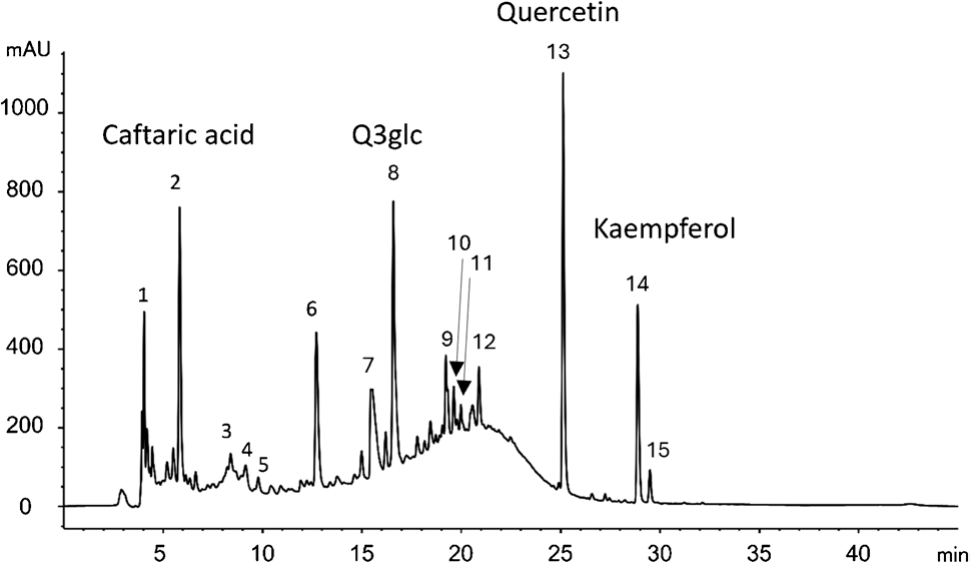
Characteristic HPLC chromatogram of extracts obtained from the grape peels obtained from the pomace recorded at 360 nm. Peak numbers are identified in Table 2. Abbreviation: Q3glc, quercetin 3-O-glucoside

Figures 1 and 2 show representative chromatograms of anthocyanins (520 nm) and flavonols and phenolic acids (360 nm) from the grape peels obtained from the pomace samples extracted using the conventional method with acidified methanol. Tentative identifications are shown in Table 2.

The obtained anthocyanin and flavonol profiles were in good agreement with the literature [25, 38, 39]. Mainly five anthocyanin-*O*-glucosides (delphinidin, cyanidin, petunidin, peonidin, and malvidin), one caffeoylglucoside, and two coumaroylglucoside derivatives were observed, with Mv3glc (peak 5 in Fig. 1) being the most abundant. In the case of flavonols, quercetin (peak 14 in Fig. 2) and its derivatives were the majority compounds.

No relevant differences were observed in the qualitative phenolic profiles obtained with the distinct NADES used for extraction. However, compared to methanol, some NADES exhibited higher selectivity towards *p*-coumaroylglucosides and phenolic acids, as highlighted by Dai et al. [17] and Panić et al. [25]. On the other hand, no interference from the NADES components with the chromatographic analysis was observed, as also shown by Dai et al. [17].

**Table 2:** Phenolic compounds identified in the grape peels obtained from the pomace

Peak	RT (min)	*λ*_max_ (nm)	[M]^+^ (*m/z*)	MS^2^ (*m/z*)	Tentative identification
**Anthocyanins**					
1	19.9	524	465	303	Delphinidin 3-*O*-glucoside
2	23.4	516	449	287	Cyanidin 3-*O*-glucoside
3	24.5	526	479	317	Petunidin 3-*O*-glucoside
4	28.1	524	463	301	Peonidin 3-*O*-glucoside
5	29.1	531	493	331	Malvidin 3-*O*-glucoside
6	42.2	535, 311 sh	655	331	Malvidin-3-*O*-caffeoylglucoside
7	44.3	502, 318 sh	637	331	Petunidin 3-*O*-*p*-coumaroylglucoside
8	46.8	532, 315 sh	611	303	Malvidin 3-*O*-*p*-coumaroylglucoside
**Phenolic acids and flavonols**					
Peak	RT (min)	λ_max_ (nm)	[M-H]^−^ (*m/z*)	MS^2^ (*m/z*)	Tentative identification
1	4.0	277	169	125	Gallic acid
2	5,8	327	311	179	Caftaric acid
3	8.4	315	295	163, 119	Coutaric acid
4	9.1	326	179	135	Caffeic acid
5	9.8	329	193	134	Ferulic acid
6	12,7	355	479	317	Myricetin 3-*O*-glucoside
7	15,4	365	301	257, 229, 185	Ellagic acid
8	16,6	354	477	301	Quercetin-3-*O*-glucuronide
			463	301	Quercetin-3-*O*-glucoside
9	19.2	357	507	345, 301	Quercetin-*O-*acetylglucoside
10	19.6	353	491	315, 301	Isorhamnetin-3-*O*-glucuronide
11	19.9	369	447	315, 301	Isorhamnetin-3-*O*-glucoside
12	20.9	372	317		Myricetin
13	25.1	371	301		Quercetin
14	28.9	367	285		Kaempferol
15	29.5	370	315	301	Isorhamnetin

### Extraction yields and NADES selection

In Table 3, the yields obtained with the different NADES for both total anthocyanin (TA) content and total flavonols (TF) content are presented. The TF and TA were calculated as the sum of the concentrations of the individual compounds quantified by HPLC-DAD. For a comparison, the table also includes the yields obtained using a conventional extraction method (methanol–0.5 N hydrochloric acid, 95:5, *v/v*).

**Table 3:** Anthocyanins and flavonols extracted from the grape peels in the pomace with the different assayed NADES

NADES	Total anthocyanins (mg/100 g dw)	Total flavonols (mg/100 g dw)
CT40	175.82	31.40
CT60	102.43	29.30
CP25	149.76	61.22
CP40	145.40	72.22
CM25	123.94	46.73
CM40	157.17	52.89
CL25	152.27	72.17
CL40	127.40	53.86
CO25	127.48	38.83
CO40	194.33	98.29
CC25	103.97	53.61
CC40	137.75	51.46
SC25	63.02	35.60
SC40	121.47	27.49
SL25	92.01	45.57
SL40	120.79	64.68
MeOH:HCl (95:5)	**95.47**	**71.88**

Under the extraction conditions used, almost all assayed NADES showed enhanced performance for extracting anthocyanins compared to MeOH:0.5 N HCl (95:5). The amounts of anthocyanins recovered ranged from 63 to 194 mg/100 g dw. Higher anthocyanin extraction yields were obtained when organic acids were used as HBDs, with choline-based NADES containing tartaric, oxalic, and malic acids showing the best performance. Good results using oxalic and tartaric acids were also reported by Loarce et al. [31]. Although sugar and alcohol-based NADES usually exhibited poorer results than organic acids due to their lower polarity [40], the anthocyanin recovery of choline-propylene glycol NADES was the fifth highest. The good performance of this NADES for anthocyanin extraction was also observed by Benvenutti et al. [41] and Loarce et al. [31]. Remarkably, the percentage of water in the choline chloride and propylene glycol solvents had no significant effect on anthocyanin recovery (Table 3).

**Fig. 3:**
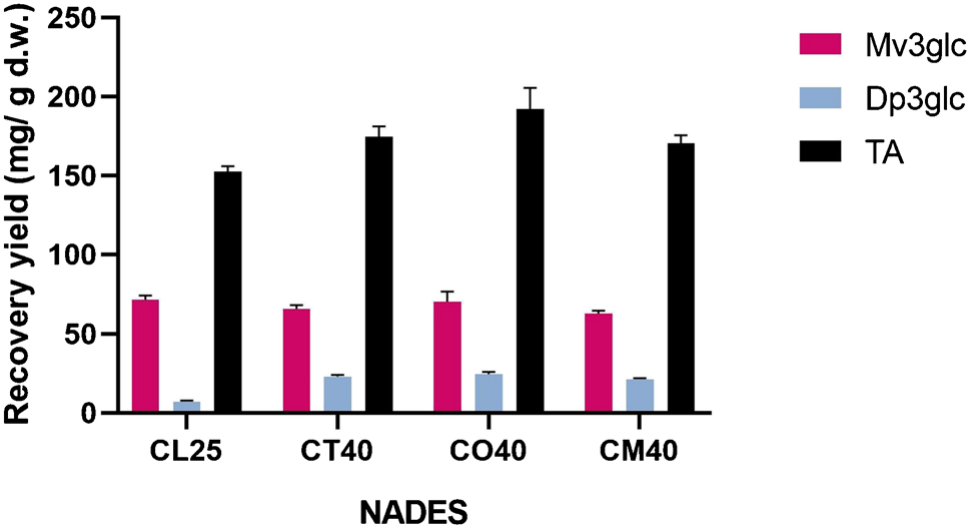
Recoveries of Mv3glc, Dp3glc, and total anthocyanins (TA) from the grape peels in the pomace obtained with different NADES. All values are mean ± standard deviation of three replicates

Another observation was that similar amounts of certain individual compounds could be extracted using different NADES, regardless of whether the total anthocyanin content obtained was higher or lower. For example, as it can be seen in Fig. 3, similar amounts of Mv3glc were obtained with solvents CL25, CT40, CO40, and CM40, and the same happened with Dp3glc and solvents CT40, CO40, and CM40, suggesting an affinity for specific target compounds by particular NADES.

Different results were obtained for flavonols, where most NADES showed less efficiency than conventional extraction with acidic methanol (Table 3). Only with choline-based NADES containing 1,2-propylene glycol and lactic or oxalic acid were similar or slightly better flavonol recoveries obtained as with methanol. Loarce et al. [42] also found good results in the pressurized hot water extraction of flavonols from grape skin using NADES containing lactic acid and alternatively oxalic acid. Ivanović et al. [28] also reported ChCl:LacA as the best solvent to extract flavonoids and phenolic acids from *Helichrysum arenarium L*. inflorescences. However, no good performance was previously reported for flavonol extraction using propylene glycol, contrary to what was observed herein. In the case of that solvent, similar to the anthocyanin extraction, the percentage of water did not affect the yield of flavonol extraction. Considering its efficiency in recovering different types of flavonoids and its versatility, this solvent seems promising for phenolic extraction.

**Fig. 4:**
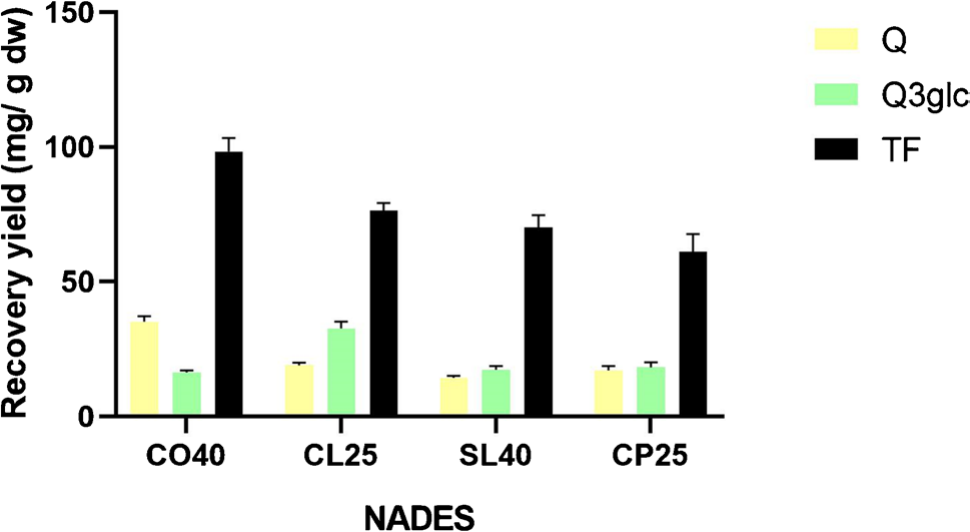
Recoveries of quercetin (Q), Qr-3-glc, and total flavonols (TF) from the grape peels in the pomace obtained with different NADES. All values are mean ± standard deviation of three replicates

The analysis of the composition of the extracts also reveals different affinities of distinct NADES towards particular compounds. For instance, different results in the extraction of quercetin and Q3glc were obtained with CO40 and CL25, with this latter solvent leading to a relatively high extraction of Q3glc despite lower amounts of total flavonols being extracted, while the opposite happened with CO40. On the other hand, similar recoveries of Q3glc were attained with CO40, SL40, and CP25 regardless of the levels of total flavonols (Fig. 4). Different selectivity depending on the solvent was also reported by Almeida et al. [43], who observed that ChCl/malic acid-based NADES extracted less than half the amount of polyphenols from olive leaves than ChCl/acetic acid combinations, but 70% more luteolin. They also found that the ChCl/malic acid solvents failed to extract tyrosol, while the other solvents did.

Taking the results together, ChCl combined with lactic acid was selected as the preferred solvent due to its good performance in the extraction of both anthocyanins and flavonols. Solvents composed of ChCl and tartaric or oxalic acid, despite their good extraction yields, were discarded because the extracts crystallized at room temperature. The results obtained also open opportunities for the application of distinct NADES in the selective extraction or enrichment of extracts when the focus is on a particular compound.

### Optimization: response surface methodology

The RSM optimization was carried out selecting ChCl and lactic acid as the extraction solvent, with water content (X_1_) and liquid to solid ratio (X_2_) as modifying factors. Water content was tested in the range from 13 to 56%, and the liquid-to-solid ratio from 13 to 56 mL/g dw. The water content was changed in order to manage viscosity while maintaining good extraction efficiency, and the liquid-to-solid ratio was modified to maximize recovery without greatly increasing costs. The extracted amounts of the majority anthocyanin (Mv3glc) and flavonol (quercetin) were considered to calculate the recovery yields. Table 4 shows the combination of levels in the 12 experiments conducted, together with the experimental recovery values for Mv3glc (Y_1_) and quercetin (Y_2_).

**Table 4:** CCCD of the independent factors (X_1_ and X_2_) for the heating extraction and experimental results for Mv3glc and quercetin (mg/100 g dw)

Run	Independent factor		Experimental response	
	X1	X2	Y1	Y2
1	20.00	20.00	73.30	42.63
2	50.00	20.00	58.52	25.69
3	20.00	50.00	75.14	38.13
4	50.00	50.00	46.05	11.47
5	13.79	35.00	78.66	40.47
6	56.21	35.00	52.04	21.86
7	35.00	13,79	63.88	38.41
8	35.00	56.21	71.94	30.87
9	35.00	35.00	78.15	21.88
10	35.00	35.00	67.38	34.71
11	35.00	35.00	69.22	31.39
12	35.00	35.00	77.22	34.26

X_1_, water content (%); X_2_, liquid-to-solid ratio (mL/g); Y_1_, Mv3glc (mg/100 g dw); Y_2_, quercetin (mg/100 g dw)

Four data fitting models were tested (see the “Optimization of the extraction methodology by response surface methodology (RSM)” section). Analysis of variance (ANOVA) was used to assess the statistical significance of the different models. According to the results of the regression analyses, the simplest adjustment without lack of fit was the linear, and the response and relationship between the two independent factors were expressed with the following equations:

$${Y}_{1}=91.18-0.68\cdot {X}_{1}+{6.40\cdot 10}^{-3}\cdot {X}_{2}$$

$${Y}_{2}=60.19-0.59\cdot {X}_{1}-0.24\cdot {X}_{2}$$

where *X*_1_ and *X*_2_ represent the water content and the liquid-to-solid ratio, respectively, and *Y*_1_ and *Y*_2_ represent the recovery of Mv3glc and quercetin, respectively.

**Fig. 5:**
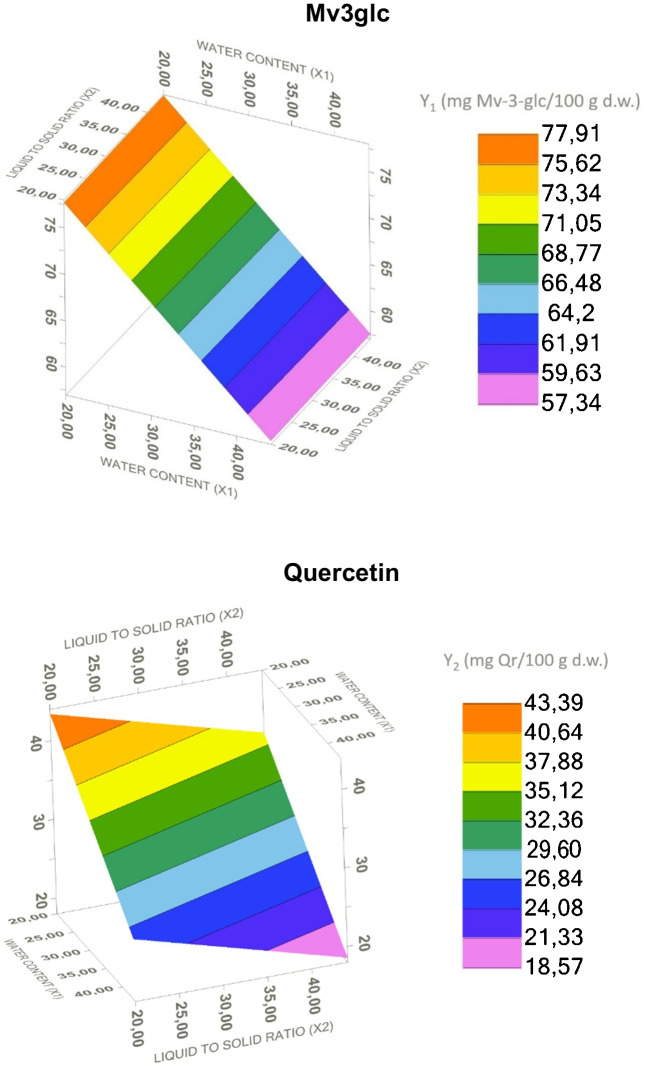
Response surface showing the combined effects of water percentage and liquid-to-solid ratio on the content of Mv3glc and quercetin

In the first equation, the model obtained was significant (*p*-value 0.0068) and fitted the experimental data adequately; the lack of fit value was 0.34. In the second equation, the model obtained was also significant (*p*-value 0.0013) and fitted the experimental data with a lack of fit of 0.78. The ANOVA of the two models showed that water content has a significant effect on both Mv3glc (*p*-value 0.0021) and flavonols (*p*-value 0.0007) extraction. The liquid–solid ratio had no significant effect on the extraction of Mv3glc (*p*-value 0.9687), but a moderate effect on the extraction of quercetin (*p*-value 0.0613). Figure 5 represents the response surface for the recovery of both compounds when water content (*X*_1_) is plotted against the liquid to solid ratio (*X*_2_).

As it can be observed in the response-surface plot, the amount of Mv3glc recovered decreased with increasing water content. The positive effect of reducing the water content below 25% in NADES based on organic acids was also observed by Bosiljkov et al. [24] and Dai et al. [44] and can be explained by the changes in polarity. Working with low water content can be advantageous since anthocyanins are less stable in NADES with higher water content [17, 18]. However, in this case, there was no significant effect of changing the liquid-to-solid ratio. As for quercetin, the response-surface plot also reveals that lower water content favours extraction. Additionally, it was noted that a solid-to-liquid ratio above 30 mg/mL reduced quercetin extraction.

Considering the models and the results obtained, the water percentage should be between 20 and 25% and the liquid-to-solid ratio should not exceed 30 mL/g (due to its influence on flavonol extraction). Therefore, the optimum point was set at 20% water and a liquid-to-solid ratio of 30 mL/g. This combination maximizes the extraction of anthocyanins and flavonols while reducing the amount of solvent needed. The highest extractability of phenolics from *Eugenia uniflora* L. was also obtained by Aguiar et al. [45] using ChCl in 20% water. The amounts of anthocyanins and flavonols obtained experimentally under the optimized conditions were 169 and 78 mg/100 g dw, respectively. Similar amounts were obtained by Panić et al. [25] and Loarce et al. [42] for the recovery of anthocyanins from grape pomace (180 and 170 mg/100 g dw, respectively), while in the case of flavonols, the amounts obtained herein are much higher than those reported by Loarce et al. [42] (19 mg/100 g dw). Nevertheless, it is important to take into account that the characteristics of the pomace (grape variety, winemaking process, etc.) could also influence the phenolic composition of the matrix and therefore the amounts extracted.

### NADES selectivity

**Fig. 6:**
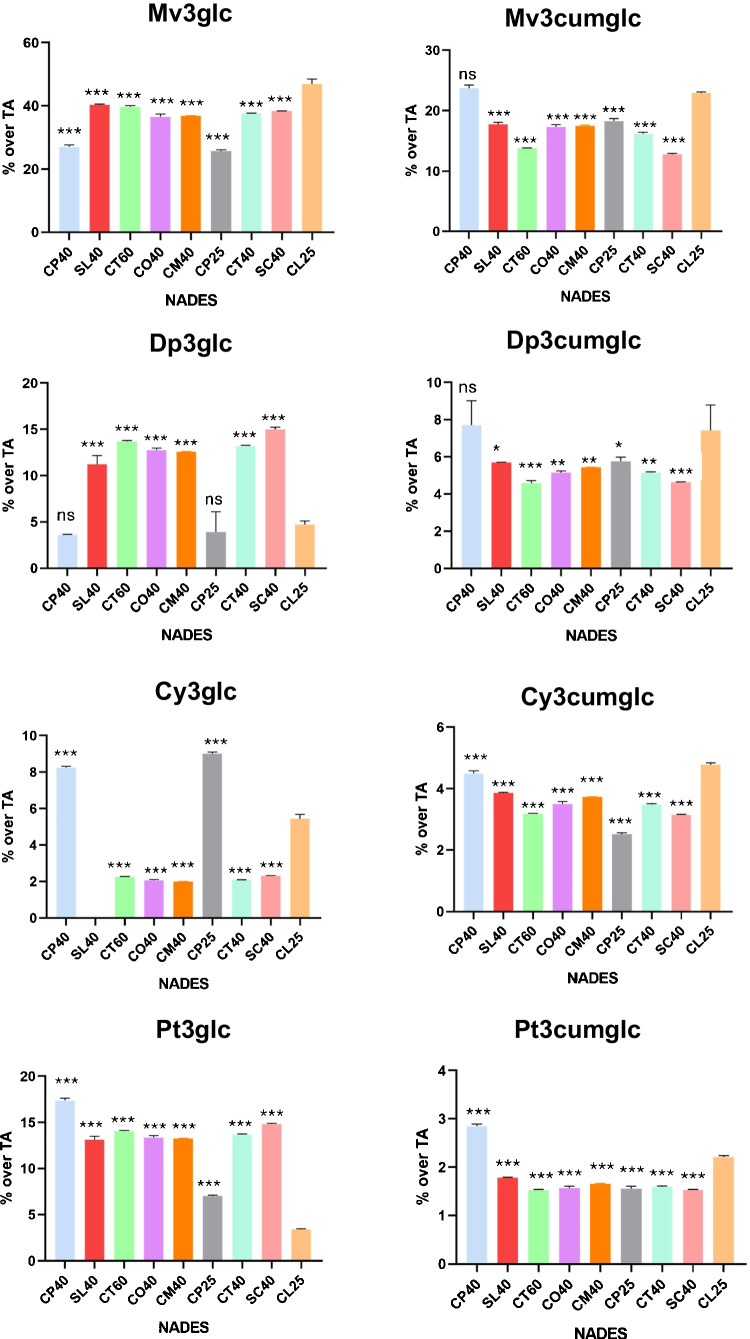
Percentages of different anthocyanins in the extracts obtained with different NADES. All values are presented as mean ± standard deviation of three replicates. Significance refers to the comparison with the solvent chosen for the optimization (CL25), with *p*-value of 0.12 (ns), 0.033 (*), 0.002 (**), < 0.001 (***)

Anthocyanins were the most abundant phenolic compounds in the extracts of grape peels obtained with all NADES. However, as discussed above, different selectivity of the assayed NADES towards different compounds was observed. Figure 6 shows the percentages of the individual anthocyanins obtained with the distinct assayed NADES in relation to the content of total extracted anthocyanins. Malvidin derivatives were the predominant compounds, with Mv3glc being always the more abundant, although its percentages varied between 30 and 50% of the total anthocyanin content, depending on the NADES. CL25 (ChCl/lactic acid, 1:2, 25% water) was the best solvent for Mv3glc extraction, while CP25 (ChCl/propylene glycol, 1:2, 25% water) behaved as the poorest one; CL25 also performed well for the extraction of coumaroylglucosides. By contrast, CP25 and CP40 (ChCl/propylene glycol, 1:2, 40% water) led to the highest extraction of Cy3glc. In general, CP40 showed to be a good solvent for most anthocyanins, except Dp3glc, for which the best results were obtained with SC40 (sucrose/citric acid, 1:1, 40% water).

**Fig. 7:**
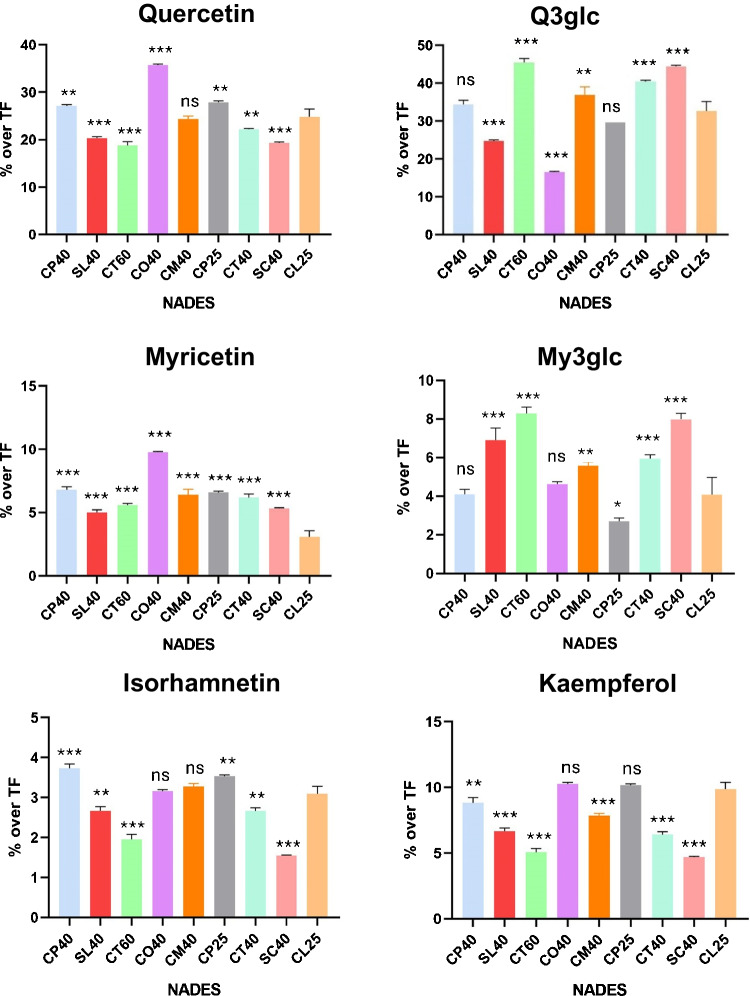
Percentages of target compounds in relation to total flavonol content (TF) in the extracts obtained with different NADES. All values are presented as mean ± standard deviation of three replicates. Significance refers to the comparison with the solvent chosen for the optimization (CL25), with *p*-value of 0.12 (ns), 0.033 (*), 0.002 (**), < 0.001 (***)

Regarding flavonols, the percentages of extraction of the individual compounds with different NADES were even more pronounced than for anthocyanins (Fig. 7), with CO40 (ChCl/oxalic acid, 1:1, 40% water) and CP25 behaving well for aglycones in general, while they were not as good for flavonol glycosides, for which CT60 (ChCl/tartaric acid, 1:1, 60% water) and SC40 seemed better.

All in all, these findings underscore the critical importance of selecting the most suitable solvent system depending on the phenolic profile of the matrix and the aim of the process, i.e. whether to maximize total phenolic extraction or to selectively recover certain compounds of interest. To the best of our knowledge, no previous study has systematically evaluated and compared the selectivity of a broad panel of NADES towards individual anthocyanins and flavonols in grape pomace. Some prior works have reported the enhanced extraction of specific compounds with certain NADES formulations. However, those observations have been limited to a few phenolics, while our study provides a comprehensive overview of the preferential enrichment of particular compounds depending on the NADES composition. For instance, CL25 proved particularly efficient for extracting Mv3glc and coumaroylglucosides, whereas SC40 was the best option for Dp3glc and CP40 favoured the extraction of anthocyanins in general. This profiling of extraction patterns highlights the potential of NADES not only as green solvents but also as selective extraction tools. Such selectivity opens up new opportunities for the targeted enrichment of bioactives, which could be advantageous for the development of functional ingredients, nutraceuticals, or natural additives with tailored compositions. Ultimately, this work reinforces the need to move beyond the assessment of total phenolic content and to consider the selective compositional profile when designing and optimizing green extraction protocols.

### Antioxidant and antimicrobial activity

To further explore the potential applications of the extracts, the antioxidant activity of those obtained with the nine most promising NADES was assessed using the FRAP and ABTS assays. The first one evaluates the reducing ability, and the second the radical scavenging capacity. The Folin-Ciocalteu reagent, although commonly used as a method for the analysis of total phenolic compounds (TPC), actually determines the reducing capacity of a sample, so that it can also be used as a measure for the antioxidant activity. The results are shown in Table 5.

**Table 5:** Antioxidant activity of NADES extracts of grape peels obtained from the pomace assessed by different methods

Solvent	TPC (mg GAE/mL)	FRAP (mM TE)	ABTS (mM TE)
CP40	0.39 ± 0.05	5.93 ± 0.54	18.76 ± 2.08
SL40	0.72 ± 0.05	5.37 ± 0.58	16.37 ± 1.55
CT60	0.44 ± 0.01	7.12 ± 0.59	19.63 ± 1.84
C040	1.10 ± 0.19	11.79 ± 1.57	34.71 ± 3.17
CM40	0.50 ± 0.08	4.48 ± 0.29	26.55 ± 1.06
CP25	0.61 ± 0.14	7.36 ± 0.53	25.38 ± 2.14
CT40	0.54 ± 0.01	6.87 ± 0.73	26.44 ± 3.02
SC40	0.57 ± 0.03	1.42 ± 0.04	9.85 ± 0.66
CL25	0.47 ± 0.03	7.27 ± 0.54	19.50 ± 1.18

*GAE* gallic acid equivalents, *TE* Trolox equivalents

The results are presented as mean value ± standard deviation

As it can be seen in the table, the Fe^2^⁺ ion reducing capacity of the extracts ranged from 1.42 to 11.79 mM TE, with the highest values observed for CO40 and the lowest for SC40. The ability to scavenge ABTS⁺ radicals ranged from 9.85 to 34.71 mM TE, with CO40 (26.95 mM TE) and CM40 (26.55 mM TE) showing the strongest activity, and SC40 (3.11 mM TE) again the weakest.

In general, a similar trend was observed between FRAP and ABTS results, although not in all cases. Notably, the extract obtained with the CM40 solvent (ChCl/malic acid, 1:1, 40% water) exhibited one of the lowest values in the FRAP assay but one of the highest in ABTS, which could be related to the fact that these methods evaluate different antioxidant mechanisms (reducing power vs. radical scavenging), and therefore not all phenolic compounds behave similarly in both assays. When a statistical analysis (Pearson correlation) was performed, no significant correlation was observed between the TPC and antioxidant activity results obtained by either FRAP or ABTS. Although the Folin-Ciocalteu assay is commonly used to estimate the total phenolic content (TPC), it actually reflects the total reducing capacity of the sample, as the reagent can react with non-phenolic reducing compounds, such as ascorbic acid, sugars, or amino acids [46]. Theoretically, a correlation between TPC and antioxidant capacity measured by FRAP could be expected since both methods are based on redox reactions. However, the lack of correlation observed in our study may be explained by the different chemical principles of the two assays. The Folin-Ciocalteu method operates under basic conditions and has a broader reactivity towards reducing substances, while the FRAP assay works at acidic pH and is more selective, primarily detecting compounds with specific structural features such as ortho-dihydroxy groups [47]. Therefore, the reducing agents quantified by Folin–Ciocalteu do not necessarily contribute equally to the antioxidant capacity measured by FRAP. In contrast, a very strong positive correlation (*p* < 0.008) was found between the results of the FRAP and ABTS assays, suggesting that despite their different mechanisms, both methods are consistent in ranking the antioxidant potential of the extracts.

Although NADES themselves do not exhibit antiradical activity, the type of NADES could also have some influence on the determined activities, as organic acids could show metal chelating properties. Indeed, the pronounced antioxidant activity of acidified NADES is evident in the ABTS assay, an effect also reported by other authors [48, 49]. In the case of the FRAP assay, a slight increase in reducing capacity was observed for solvents containing tartaric acid. Differences in the antioxidant activity and TPC of extracts obtained using different NADES have also been reported by other authors. For instance, Božović et al. [50], in sour cherry kernels extracts, found that those obtained with choline chloride/glucose (1:2) led to the highest TPC, while the greatest antioxidant activities in the DDPH and FRAP assays were achieved using choline chloride/citric acid (1:2) and tartaric acid/xylitol (1:2), respectively.

In addition to their antioxidant properties, phenolic compounds are also known for their antimicrobial potential. For this reason, the antimicrobial activity of the extracts was evaluated against three Gram-positive (*Staphylococcus aureus*, *Bacillus subtilis*, and *Listeria innocua*) and two Gram-negative bacteria (*Escherichia coli* and *Proteus mirabilis)* of relevance in the food industry. The results are shown in Table 6.

**Table 6:** Antimicrobial activity (MIC values*) of NADES extracts of grape peel obtained from the pomace

*Sample*	Gram-positive bacteria			Gram-negative bacteria	
	*Staphylococcus aureus*	*Bacillus subtilis*	*Listeria innocua*	*Escherichia coli*	*Proteus mirabilis*
CP40	-	-	-	-	-
SL40	25.0	-	50	12.5	25.0
CT60	50.0	-	50	25.0	50.0
C040	50.0	100	100	25.0	50.0
CM40	25.0	-	50	12.5	100
CP25	-	-	-	-	-
CT40	25.0	-	100	25.0	100
SC40	25.0	-	100	25.0	100
CL25	25.0	-	25.0	25.0	25.0

*MIC values (minimum inhibitory concentrations) are expressed as the lowest percentage of the NADES-based extract at which microbial growth is visibly reduced

The explored extracts exhibit some variable antimicrobial activity against the bacteria tested. Among Gram-positive bacteria, the lowest MIC value (25.0%) against *Staphylococcus aureus* was observed for the extracts SL40, CM40, CT40, SC40, and CL25, while for *Listeria innocua*, the extract with the strongest inhibitory effect was CL25. Interestingly, this extract had also been selected as the most effective for the combined extraction of anthocyanins and flavonols, which reinforces its suitability for food-related applications, given its dual antioxidant and antimicrobial functionality. In the case of *Bacillus subtilis*, only the CO40 extract showed some inhibition of bacterial growth. In gram-negative bacteria, the lowest MIC value (12.5%) for *Escherichia coli* was found for extracts SL40 and CM40, whereas for *Proteus mirabilis*, the lowest MIC (25.0%) was observed for CL25 and SL40. No bacterial inhibition was detected in extracts based on choline chloride and propylene glycol, which supports the enhancing effect of acidified NADES, as previously reported by other authors [48, 49, 51]. It must be, however, noted that the antimicrobial activity of the obtained extracts did not differ from that of the corresponding pure NADES (data not shown), indicating that the observed effects were primarily attributable to the solvent itself rather than the extracted phenolic compounds. This finding would be in agreement with the observations of other authors [49, 51] who reported that NADES could have significantly greater antimicrobial activity than the extracted components. Although the antimicrobial activity can be attributed to the presence of NADES in the extracts, it is noteworthy that no such activity was observed in the methanolic extracts under the conditions tested. This suggests a potential added value of NADES-based extracts, which not only enable the recovery of antioxidant compounds but also provide a certain degree of antimicrobial protection. This dual functionality, resulting from the solvent system, could be particularly advantageous in developing natural additives for the food industry, where oxidative stability and microbial safety are critical.

## Conclusions and future perspectives

The effectiveness of a range of NADES for extracting anthocyanins and flavonols from grape peels obtained from winemaking pomace has been explored. The effects of two operational parameters, namely water content and solvent-to-solid ratio, on the extraction of both compound families were examined. While all the assayed NADES performed well for the extraction of anthocyanins compared with conventional extraction with acidified methanol, they were less efficient for flavonols. Overall assessment of the results pointed at the system ChCl/lactic acid, 1:2 containing 25% water (CL25) as a satisfactory compromise alternative. Interestingly, this extract showed relatively high antioxidant activity when evaluated by the FRAP assay (even though more moderate when evaluated by the antiradical ABTS method), and this NADES also exhibited the strongest antimicrobial effect against *Listeria innocua*, reinforcing its potential for food applications.

Nevertheless, marked differences were found in the affinity of different NADES towards distinct phenolic compounds, which could be exploited for the rational selection of the most suitable solvent depending on the phenolic profile of the matrix or when searching for particular target compounds. Techniques such as ultrasound- or microwave-assisted extraction may also be considered to further improve efficiency and reduce energy consumption.

Despite their potential, the application of NADES-based extracts remains complex. Their physical and chemical characteristics make them unsuitable for freeze-drying, and drying generally requires the addition of bulking compounds such as maltodextrins, which may compromise the bioactive properties of the extracts [52]. Alternatively, additional purification through adsorption resins allows the recovery of phenolic compounds but requires the use of organic solvents such as ethanol, thereby increasing costs and reducing process sustainability [53, 54].

In light of these challenges, the most sustainable and realistic strategy may not lie in overcoming purification hurdles, but in considering NADES-based extracts as “ready-to-use” liquid formulations. Extracts of this kind, prepared from food-grade components, have the potential to provide functional antioxidant and antimicrobial properties, thus replacing synthetic additives and meeting the demand for clean-label natural ingredients. Nevertheless, while the individual components of NADES (e.g. choline chloride, sugars, organic acids) are generally recognized as safe, the toxicity of the mixtures might not be straightforward, depending on their formulation, molar ratio, dose, and route of administration. The evidence in this regard is inconsistent: while some studies have reported no toxic effects in animals [52, 55, 56], others have observed increased toxicity or even mortality [57–59]. Research has identified the hydrogen bond donor, water content, and physicochemical properties of the solvents as critical determinants of their toxicological behaviour [58, 59]. This underscores the necessity for a thorough, case-by-case assessment of NADES safety. In the end, rigorous toxicological studies will be essential to ensure their safety prior to any practical application in the food industry.

## Data Availability

All data is available from the corresponding author upon reasonable request.
